# Do Mutations Turn p53 into an Oncogene?

**DOI:** 10.3390/ijms20246241

**Published:** 2019-12-11

**Authors:** Consuelo Pitolli, Ying Wang, Mara Mancini, Yufang Shi, Gerry Melino, Ivano Amelio

**Affiliations:** 1 Department of Experimental Medicine, TOR, University of Rome Tor Vergata, 00133 Rome, Italy; cp674@mrc-tox.cam.ac.uk (C.P.); mara.mancini@hotmail.it (M.M.); gm614@mrc-tox.cam.ac.uk (G.M.); 2 MRC Toxicology Unit, University of Cambridge, Pathology Building, Tennis Court Road, Cambridge CB2 1PQ, UK; 3 CAS Key Laboratory of Tissue Microenvironment and Tumor, Institute of Nutrition and Health, University of Chinese Academy of Sciences, Chinese Academy of Sciences, Shanghai 100012, China; yingwang@sibs.ac.cn (Y.W.); shiyufang2@gmail.com (Y.S.); 4 IDI-IRCCS, Biochemistry Laboratory, 00167 Rome, Italy; 5 Institutes for Translational Medicine, Soochow University, Suzhou 215006, China

**Keywords:** TP53, mutant TP53, gain of function, oncogenic

## Abstract

The key role of *p53* as a tumor suppressor became clear when it was realized that this gene is mutated in 50% of human sporadic cancers, and germline mutations expose carriers to cancer risk throughout their lifespan. Mutations in this gene not only abolish the tumor suppressive functions of p53, but also equip the protein with new pro-oncogenic functions. Here, we review the mechanisms by which these new functions gained by *p53* mutants promote tumorigenesis.

## 1. Introduction

*TP53* (Tumor Protein P53) is among the most extensively studied human genes [1,2]. The main explanation for this interest is its key role in preventing tumor development. Indeed, the transcription factor p53 is the principal mediator of cellular responses to several stressors, such as DNA damage, oncogene activation, nutrient deprivation, and hypoxia [3,4]. In unstressed cells, p53 activity is negatively regulated by MDM2 (Mouse Double Minute 2), which binds to p53 and promotes its proteasomal degradation [5,6,7,8]. Interestingly, *MDM2* itself is a p53 target gene [9,10,11,12]. Thus, p53 and MDM2 establish an autoregulatory negative feedback loop, to maintain low cellular p53 levels in the absence of stress. In response to a stress stimulus, both p53 and MDM2 undergo post-translational modifications that block their interaction. As a result, MDM2-mediated inhibition is alleviated, leading to p53 accumulation and activation [13]. The activation of p53 results in three major outcomes: growth arrest, DNA repair, and apoptosis. Growth arrest causes a temporary arrest of cell cycle progression, enabling the cell to correct damaged DNA, and prevent the replication of damaged DNA and the transfer of the genetic aberrations to daughter cells. In addition to inducing cell cycle arrest, p53 also promotes DNA repair [14,15,16,17]. Once DNA repair is complete, the cell cycle resumes. In contrast, if the cell has severe DNA damage that is unable to be repaired, p53 eliminates the cell by inducing programmed cell death [18]. Thus, p53 acts as a guardian of the genome by preventing the accumulation of oncogenic mutations that could lead to tumor development [19].

The effects of p53 are mainly mediated by its transcriptional activity [20]. In particular, p53-induced cell cycle arrest involves the transcriptional activation of *CDKN1A/P21* (Cyclin Dependent Kinase Inhibitor 1A) [21,22,23,24]. During apoptosis, p53 increases the expression of a large number of genes, including *BBC3/PUMA* (Bcl-2-Binding Component 3), *PMAIP1/NOXA* (PMA-Induced Protein 1), *BAD* (BCL2 Associated Agonist Of Cell Death), *BAX* (BCL2 Associated X), *BAK* (BCL2 Antagonist), *TP53AIP1* (Tumor Protein P53 Regulated Apoptosis Inducing Protein 1), and *FAS* (Cell Surface Death Receptor) [25,26].

p53 also functions in DNA repair by transcriptionally regulating the expression of genes involved in several DNA damage repair (DDR) pathways. In vivo shRNA screens targeting p53-regulated genes demonstrate that DNA repair is a crucial mechanism in p53 suppression of tumor development [27]. p53 has been shown to exert this role at different levels, for example in Nucleotide Excision Repair (NER), p53 induces *DDB2* (Damage Specific DNA Binding Protein 2) and *XPC* (XPC Complex Subunit), two components of the NER machinery [28]. p53 can also regulate the transcription of Base Excision Repair (BER) genes such as *OGG1* (8-Oxoguanine DNA Glycosylase) [29] and *MUTYH* (MutY DNA Glycosylase) [30], which encode for an 8-oxoguanine glycosylase and an adenine DNA glycosylase, respectively. p53 influences BER also though its ability to regulate the expression of 3-methyladenine (3-MeAde) DNA glycosylase, the first enzyme in the BER pathway [31], and APE1, an apurinic/apyrimidinic (AP) endodeoxyribonuclease [32]. p53 also synergizes to the transcription factor c-jun to regulate the transcription of the *MSH2* (MutS Homolog 2) gene, encoding a component of the DNA mismatch repair system (MMR) [33].

p53 also acts in DNA Double-Strand Break Repair (DNA-DSB). Nonhomologous End Joining (NHEJ) and Homologous Recombination (HR) are the two pathways involved in DNA-DSB repair. Although several studies suggest a connection between p53 and NHEJ [34,35], the role of p53 in this process is still unclear. On the contrary, p53 has been shown to regulate HR by inducing the expression of *RAD51* (BRCA1/BRCA2-Containing Complex) [36]. In addition to these transcription-dependent functions, p53 has been shown to have transcriptional independent functions in promoting tumor suppression. For example, in the cytosol, p53 can directly bind and activate BAX. Upon activation, BAX forms homo-oligomers that are inserted in the outer mitochondrial membrane (OMM), inducing membrane permeabilization, cytochrome c release, and caspase-3 activation [37]. In the regulation of the NER pathway, p53 has been shown to interact with the helicases XPB (Xeroderma Pigmentosum, Complementation Group B) and XPD (Xeroderma Pigmentosum Complementary Group D), modulating their activities [38,39]. Furthermore, p53 stimulates BER by interacting and stabilizing DNA pol β, the main DNA polymerase involved in BER [40]. In addition, p53 modulates HR via direct interactions with RAD51 and RAD54L (DNA Repair And Recombination Protein RAD54-Like) proteins [41].

The regions of p53 that are responsible for recognizing specific p53-binding elements in the promoters of its target genes, and subsequent transcriptional activation, are well defined. The p53 protein comprises a transactivation and a proline-rich domain (residues 1 to 43 and 61 to 94, respectively) located at the N-terminus, a central DNA-binding domain (DBD) (residues 110 to 286), and a tetramerization domain (TD) and regulatory region (residues 326 to 355 and 363 to 393, respectively), located at the C-terminus. Among these domains, the TD allows the oligomerization of the protein, and the formation of a tetrameric complex that represents the active conformation of p53 [42].

Although researchers initially postulated that p53 tumor suppressor activity was mainly mediated through induction of cell cycle arrest and apoptosis, additional mechanisms have more recently emerged [43]. Indeed, p53 also controls additional cellular processes that are potentially important for suppressing tumor formation, such as the metabolism, autophagy, ferroptotic cell death, and stemness [44,45,46].

## 2. p53 Mutations: One Gene Different Proteins

Given the fundamental role of p53 in restricting tumor formation, its inactivation is commonly identified in human cancers [47]. Somatic mutations in p53 occur in over half of all human cancers, while germline p53 mutations that abolish its function are observed in a hereditary form of cancer, known as Li-Fraumeni syndrome [48]. Further supporting the role of p53 in preventing tumor development, Trp53 knockout mice show a high predisposition to tumor formation [49].

**Figure 1:**
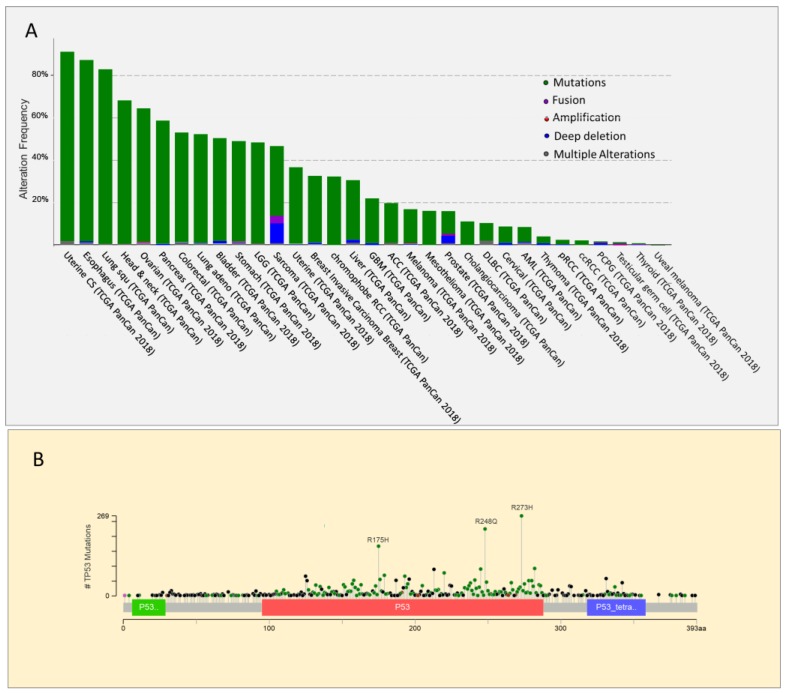
Analysis of TP53 genetic alterations. using cBioPortal data. (**A**). Frequency and type of TP53 alterations in different cancer types. Alterations in corresponding color codes are as follows: mutations (green), fusions (purple), amplifications (red), deep deletions (blue), and multiple alterations (grey). Source The cBio Cancer Genomics Portal is an open platform for exploring multidimensional cancer genomics data (cbioportal.org). (**B**) Distribution and frequency of different types of mutations along the p53 protein. Mutation types and corresponding color codes are as follows: missense mutations (green), truncating mutations (black), inframe mutations (red), and other mutations (Pink).

*TP53* gene has an unusual mutational pattern. Indeed, the gene is not frequently deleted but is mainly subject to mutations, the majority of which are missense mutations located in the DNA binding domain [50] (Figure 1). Within this region, the most frequent mutations, known as hot spots, are divided into two categories: conformational mutations (e.g., R175H) that lead to structural changes in the binding domain, and contact mutations (e.g., R273H), that alter the ability of the protein to bind DNA [51]. In both cases, these mutations alter the interaction of p53 with its consensus DNA-binding sequence, impairing the activation of p53 target genes involved in suppressing tumor growth. The frequency of somatic and germline hot spot p53 mutations in human cancer is reported in Table 1. Very high levels of p53 mutant proteins accumulate in tumors because of their inability to induce MDM2 expression [52]. However, p53 mutants have not only lost the tumor suppressor function of wild type p53 associated with its transcriptional activity [53,54], but also exert a dominant negative effect on the co-expressed wild type protein. Indeed, mutant p53 heterodimerizes with wild type p53 to form complexes that impair its function [55,56,57]. p53 mutations are usually followed by the deletion of the remaining wild type *TP53* allele. This phenomenon, known as loss of heterozygosity (LOH), suggests that despite the dominant negative effect exerted by p53 mutants, the complete loss of wild type p53 provides cancer cells with a selective advantage [58]. Indeed, according to a recent in vivo study, p53 LOH is required for mutant p53 stabilization, and the execution of additional oncogenic functions. Indeed, mutant p53 proteins can also acquire novel pro-oncogenic properties, an effect known as gain of function (GOF). In particular, mouse tumors that undergo to p53 loss of heterozygosity at a high frequency exhibit stabilization of the mutant p53 protein, and an accelerated tumor onset compared with p53+/− tumors. In contrast, in mouse tumors in which wild type p53 LOH rarely occurs, the mutant p53 protein is not stabilized, and GOF activity is not observed [59].

GOF in p53 is supported by evidence that mice expressing p53 R172H or R270H mutants (equivalent to human R175H and R273H) develop a greater number of metastatic tumors than p53−/− mice [60,61], and by the observation that patients with Li-Fraumeni syndrome carrying p53 missense mutations are characterized by earlier tumor development, than patients with a *p53* deletion [62]. Interestingly, different p53 mutant proteins are associated with different GOF effects. Indeed, distinct cancer phenotypes were observed in knock-in mice harboring different p53 mutants. In particular, p53R270H/+ mice had an increased incidence of carcinomas and B cell lymphomas compared to p53+/−. In contrast to the frequent carcinomas in p53R270H/+ mice, p53R172H/+ mice developed mainly osteosarcomas [61]. In addition, human *p53* knock-in (hupki) mice harboring the hot spot mutation R248Q, display an accelerated tumor onset and shorter survival, compared to *p53*-null mice. However, homozygous G245S hupki mice had similar overall survival and tumor spectrums to their *p53*-null counterparts, further supporting that different *p53* mutants have variation in their GOF activities [63]. Consistently, Li-Fraumeni syndrome patients with different TP53 missense mutations showed different tumor spectra. Specifically, the median age at diagnosis is 19.5 years for patients with mutations at the R248Q codon, compared to 30 years for patients with a nonsense *p53* mutation. However, in Li-Fraumeni patients with R248Q mutation, the disease occurs on average at 19.5 years, in patients with G245S *TP53* mutations, the disease is diagnosed at a median age of 30.5 years [64].

Depending on the tumor type, *p53* inactivation (deletions and/or mutations) can occur at different steps of the malignant progression. In many solid tumors (colorectal cancer, pancreatic cancer, and breast cancer for example), *p53* mutations are among the later steps of the tumorigenesis, in other contexts, these genetic events can occur at early stages (e.g., in esophageal carcinoma) [65,66,67]. Definitive studies on the genetic evolution of cancers are only recently emerging. In pancreatic ductal adenocarcinoma (PDAC) genetic evolution has been quite well defined. PDAC arises from precursor lesions (PanINs) that progressively evolve toward the highly invasive and metastatic PDACs in which *p53* is mutated in 75% of cases. Concurrently, with SMAD4 (SMAD Family Member 4 inactivation), inactivating mutations of *TP53* occur only during the late phase of pancreatic carcinogenesis, following early (activating mutations in KRAS Proto-Oncogene) and intermediate (inactivating mutations of Cyclin Dependent Kinase Inhibitor 2A) genetic events [68]. The acquisition of *p53* mutations during the advanced stage of the disease might confer cancer cells high genomic instability, a metastatic phenotype, and progression toward very aggressive PDACs.

To date, mutant p53 has been shown to promote oncogenic cellular changes by interacting with other transcription factors (enhancing or impairing their transcriptional activity) or with chromatin-modifying complexes, leading to alterations in the cellular transcriptional profile. It has been suggested that missense mutations at different sites may impose specific conformational changes that can influence the affinity of each p53 mutant protein for different binding partners. Thus, several p53 mutants may have the ability to interact with different proteins resulting in the transactivation of different set of target genes, and variations in cellular phenotypes [69]. Through these mechanisms, p53 mutants have been shown to affect multiple aspects of cellular behavior and phenotypes, such as metabolism, invasion, migration, and proliferation.

## 3. Mutant p53 and Cancer Therapy Resistance

The major aim of cancer therapy is to inhibit cell proliferation and promote cell death. Interestingly p53 mutant expression has been associated with chemoresistance in breast cancer [70], ovarian cancer [71], lung cancer [72], and gastric and colorectal cancers [73]. It is not only the loss of the key pro-apoptotic function of wild type (wt) p53 to confers chemotherapy resistance, but also GOF effects exerted by mutant p53 proteins to contribute to drug resistance. For example, p53 mutants stimulate the expression of ABCB1 (ATP Binding Cassette Subfamily B Member 1), an ATP-binding cassette (ABC) transporter, mediating the efflux of drugs from cells in a ATP-dependent manner, conferring multi-drug resistance (MDR) [74]. Interestingly wt p53 exerts the opposite effect on ABCB1 expression compared to p53 mutants [75]. Interestingly p53 mutants block Ataxia Telangiectasia Mutated (ATM)-dependent activation of the DNA damage response (DDR) through the disruption of the MRE11-RAD50-NSB complex. On the other hand, mutant p53 stimulates the activity of the enzyme poly (ADP ribose) polymerase 1 (PARP1), allowing tumor cells to survive in the presence of high levels of DNA damage [76,77]. In addition to the induction of chemoresistance, both in vitro [78] and in vivo [79,80] studies have shown that p53 mutants are able to induce resistance to radiotherapy. Moreover, in several human cancers, p53 mutants are associated with reduced radiosensitivity and worse prognosis [73,81].

## 4. Effect of Mutant p53 GOF on p53 Family Members: Tumor Invasion and Metastasis

**Figure 2:**
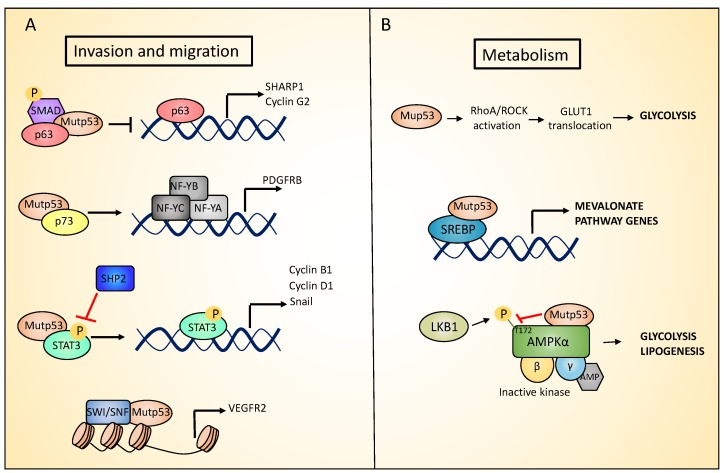
Mutant p53 gain of function (GOF) in metabolism and invasion. (**A**). Mutant p53 proteins promote invasion and migration by inhibiting p63 and p73 function, enhancing STAT3 (Signal Transducer And Activator Of Transcription) signaling and vascular endothelial growth factor receptor (VEGFR) expression. (**B**). Mutant p53 proteins promote glycolysis by upregulating glucose transporter GLUT1 translocation to the plasma membrane. In addition, mutant p53 proteins stimulate the mevalonate pathway through a physical interaction with SREBPs (Sterol Regulatory Element Binding Transcription Factors), and enhance lipogenesis by inhibiting AMP-activated protein kinase (AMPK) signaling.

As discussed above, some solid tumors, such as PDAC, accumulate mutations in *p53* in the later stages of the tumor progression and this correlates with the acquisition of an invasive/metastatic phenotype. Several possible mechanisms have been described to explain p53 mutant’s ability to drive metastasis, such as involving the p53 homologs, p73 and p63 [82,83]. Given their high structural similarity, p63 and p73 bind and activate many p53 target genes to regulate cell cycle arrest and apoptosis, in response to cellular stress. Notably, p63 and p73 homo and heterodimerize with each other, but do not interact with wild type p53. On the contrary, the conformational mutant p53R175H has been shown to aggregate to the family members p63 and p73, through an interaction that involves its DNA binding domain (DBD) and the C-terminal transactivation inhibitory (TI) domain of both p63 and p73 α-isoforms. Supporting this hypothesis, the TAp63α that in normal conditions acquires a closed conformation in which the TI domain is inaccessible, does not interact with p53R175H [84]. Through this co-aggregation mechanism, mutant p53 proteins may exert a dominant negative effect on p63 and p73, inhibiting their functions [85,86,87]. In particular, the formation of mutant p53/p63 or mutant p53/p73 complexes has been shown to promote invasion through several mechanism. The inhibition of p63 function mediated by the interaction with mutant p53 (both the R175H and R273H mutants) represses *SHARP-1* (Basic Helix-Loop-Helix Family Member E41) and *Cyclin G2* expression, promoting cell migration and invasion. Phospho-SMAD2 (SMAD family member 2), a component of the transforming growth factor beta (TGFβ) signaling pathway that serves as a scaffold for p53-p63 complex assembly, plays a key role in this process (Figure 2A) [88,89]. In addition, the mutant p53-dependent suppression of p63 activity increases the Rab coupling protein (RCP)-driven recycling of α5β1 integrin and EGFR (epidermal growth factor receptor) to the cell surface, leading to the activation of Rho and PKB/Akt signaling that promote cell migration and invasion [90]. In pancreatic cancer, mutant R172H p53 has been shown to interact with p73, blocking the interaction of p73 with NF-Y (nuclear transcription factor Y), which in turn induces the expression of *PDGFRβ* (platelet-derived growth factor receptor beta), which is important for maintaining a metastatic phenotype (Figure 2A) [91].

## 5. Mutant p53 Between Tumor Development and Self-Renewal

According to the cancer stem cell hypothesis, tumors are initiated and sustained by a small fraction of cells termed cancer stem cells (CSCs) or tumor-initiating cells, that have the ability to self-renew as well as to differentiate into various lineages. It is quite commonly accepted that CSCs are responsible for tumor chemoresistance and relapse, thus representing an important therapeutic target [92]. CSCs are thought to originate from normal stem cells (SCs) that underwent oncogenic genetic modifications, or from the dedifferentiation of progenitor or somatic cells that gain stem cell like characteristics and became CSCs [93]. Wt p53 has been shown to promote differentiation and restrain proliferation of stem cells. In addition, wt p53 acts as a barrier for the reprogramming of terminally differentiated cells into stem cell-like cells. Given these roles of p53 in the control of differentiation/de-dedifferentiation processes, p53 mutations could influence stem cell differentiation, participating in cancer development by facilitating CSC maintenance [94,95].

p53 mutants have been shown to facilitate the formation of CSC either by promoting oncogenic transformation of adult stem cells, or the dedifferentiation of somatic cells. In particular, humanized mouse models harboring mutant p53 show a higher number of mesenchymal and hematopoietic SCs, compared to p53-null mice [63]. Interestingly, bone and soft-tissue sarcoma, whose incidence is very high in Le-Fraumeni patients [96], has been suggested to arise from defective mesenchymal stem cells (MSCs) [97,98]. Indeed, MSCs heterozygous for mutant p53 frequently undergo to p53 LOH that result in an increase tumorigenic potential [99]. Likewise, mutant p53, by promoting human osteosarcoma cells dedifferentiation, leads to increased proliferation, invasiveness, and resistance to apoptosis [100]. A further study shows that a *Trp53^R172H^* mutation promotes the initiation and the maintenance of acute myeloid leukemia (AML), by enhancing the self-renewal property of hematopoietic stem and progenitor cells (HSPC). Mechanistically, mutant p53 exerts this GOF activity by upregulating the expression of the *Foxh1* (Forkhead Box H1) gene, which encodes a key transcription factor involved in the regulation of stem cell-associated genes [101].

## 6. Role of Mutant p53 Gain of Function in Metabolism and Hypoxia

p53 mutations have also been associated to deregulation of cellular metabolism, independently from the primary role that the wt p53 exerts. In breast cancer cells, p53 mutants stimulate the mevalonate pathway. This pathway is responsible for the production of cholesterol that in turn is required for membrane biogenesis and cell division. p53 mutants exert this function acting as coactivators of SREBPs (Sterol Regulatory Element Binding Transcription Factors) proteins (SREBP-1 and SREBP-2), that are transcription factors promoting the expression of key mevalonate pathway enzymes [102]. In particular, co-immunoprecipitation experiments show that mutant p53 interacts with SREPBs proteins (Figure 2B). Furthermore, chromatin immunoprecipitation (ChIP) analysis have identified mutant p53 binding in the proximity of SREBPs binding sites in the promoter of SREBPs target genes. SREBPs are required for mutant p53 recruitment on these promoters. Indeed, depletion or pharmacological inactivation of SREBPs partially abolishes mutants p53 recruitment on the promoters of these genes. The stimulation of the mevalonate pathway by p53 mutants is necessary to maintain the malignant state. Indeed, under 3D culture conditions, p53 mutated breast cancer cells form highly disorganized and invasive structures, that were reverted toward acinus-like structures following mutant p53 depletion. The supplementation of p53 mutated breast cancer cells in 3D culture with mevalonate pathway intermediates impairs the phenotypic reversion caused by mutant p53 downregulation. Coherently, the pharmacologic inhibition of the mevalonate pathway recapitulates the effects of knocking down mutant p53 [103,104,105,106]. Interestingly opposing mutant p53, wild type p53 represses the mevalonate pathway. In particular, the activation of wild type p53 inhibits SREBP-2 maturation, leading to the downregulation of its target genes. Wild type p53 affects SREBP-2 maturation by activating the transcription of the cholesterol transporter gene ABCA1 (ATP Binding Cassette Subfamily A Member 1), that has been reported to mediate the retrograde transport of cholesterol from the plasma membrane to the endoplasmic reticulum (RE), suppressing SREBP-2 maturation. In line with this, in a mouse model of liver cancer, the ablation of ABCA1, similarly to p53 loss, promoted tumorigenesis and was associated with increased SREBP-2 maturation [107]. Furthermore, under conditions of metabolic stress, mutant p53 increases lipid production and aerobic glycolysis (i.e., the Warburg effect) by inhibiting AMP-activated protein kinase (AMPK) signaling. AMPK is a cellular energy sensor that is activated in response to a decrease in ATP (adenosine triphosphate) and a parallel increase in AMP (adenosine monophosphate) or ADP (adenosine diphosphate) levels. After activation, AMPK increases ATP production by promoting catabolic pathways, and inhibiting anabolic processes. AMPK is a serine/threonine protein kinase heterotrimer, composed of a catalytic subunit (α), and two regulatory subunits (β and γ). In response to energy stress, AMPK activation requires the AMP or ADP binding to the γ regulatory subunit. This binding leads to conformational changes that allows the activating phosphorylation of the Thr172 residue in the AMPKα subunit by the serine/threonine kinase LKB1 [108]. Mutant p53 inhibits AMPK activation by binding the AMPKα subunit and blocking its Thr172 phosphorylation by LKB1 (Liver Kinase B1), or impairing AMPKα-LKB1 interaction (Figure 2B) [109,110].

**Figure 3:**
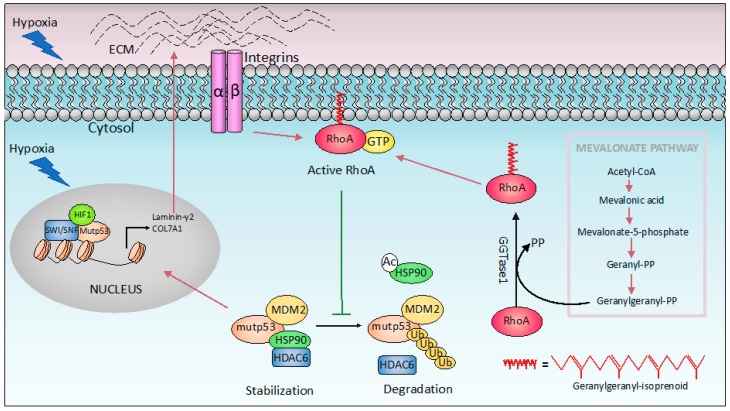
Mutant p53 GOF and hypoxia. In response to hypoxia, p53 mutant forms a complex with hypoxia-inducible factor 1 (HIF-1) that physically binds the SWI/SNF chromatin remodeling complex, promoting expression of extracellular matrix (ECM) components such as Laminin-γ2 and type VIIa1 collagen (COL7A1). On the other hand, increased ECM stiffening induces HDAC6/ Heat shock protein 90 (Hsp90)-dependent stabilization of mutant p53 from ubiquitin-mediated proteolysis, through a mechanism that involves RhoA geranylgeranylation downstream of the mevalonate pathway. The purple and green arrows indicate promotion and inhibition, respectively.

Hypoxia is a common characteristic of solid tumors. Tumor cells generally adapt to hypoxic stress by activating numerous intracellular signaling pathways that promote angiogenesis, and the acquisition of a more invasive and metastatic phenotype that allows tumor cells to survive or escape from the hypoxic environment [111,112]. Mutant p53 has been shown to promote cancer cell adaptation to hypoxia. First, mutant p53 stimulates neo-angiogenesis in tumors by increasing the production of VEGF (Vascular Endothelial Growth Factor) in bone marrow stromal cells [113], or upregulating ID4 (Inhibitor Of DNA Binding 4), that in turn increases the secretion of pro-angiogenic cytokines such as IL-8 [114]. In addition, mutant p53 induces VEGFR2 (vascular endothelial growth factor receptor 2) expression, leading to increased cellular growth. Interestingly, mutant p53 cooperates with the SWItch/Sucrose Non-Fermentable (SWI/SNF) complex to remodel the chromatin architecture at the VEGFR2 promoter (Figure 2A) [115]. Interestingly under hypoxic conditions, p53 mutant cooperates with HIF-1 (hypoxia-inducible factor 1), the master transcriptional regulator of the cellular response to oxygen deprivation, to selectively regulate the expression of specific HIF-1-responsive genes. In particular, p53 mutants interact with HIF-1, forming a complex promoting the transcription of extracellular matrix (ECM) genes such as type VIIa1 collagen (*COL7A1*) and laminin-γ2 (*LAMC2)* (Figure 3). Mechanistically, the selectivity of the p53 mutant/HIF-1 transcriptional complex on this specific subset of genes involves chromatin remodeling mediated by the SWI/SNF complex. This complex does not affect the recruitment of mutant p53/HIF-1 to the genomic regions of type VIIa1 collagen and laminin-γ2 ECM genes, but it is required for their hypoxia-dependent up-regulation. The analysis of the chromatin architecture at the type VIIa1 collagen and laminin-γ2 promoter regions, reveals the requirement of mutant p53 to maintain a more open and transcriptionally accessible status. These data suggest that mutant p53 facilities the chromatin remodeling activity of SWI/SNF at the genomic regions of these ECM genes, promoting their expression. The transcriptional activation of these ECM genes by p53 mutant/HIF-1 complex is associated with the acquisition of an aggressive phenotype both in vivo and in vitro. Indeed, while mutant p53 depletion impairs hypoxia mediated invasion and migration in non-small cell lung cancer (NSCLC), the ectopic expression of VIIa1 collagen and laminin-γ2 reverts this impairment. Similarly, in a cancer mouse model obtained by hypoxic preconditioned non-small cell lung cancer xenotransplantation into immunocompromised mice, the depletion of mutant p53 was associated with reduced tumor growth that was reverted by the overexpression of laminin-γ2 or type VIIa1 collagen. Coherently, in human NSCLC patients, the expression of these ECM genes is correlated with HIF-1 activation exclusively in patients carrying p53 mutations, and is associated with a worse prognosis [116,117].

A further study shows that the increase in ECM stiffness induces mutant p53 stabilization that is a prerequisite for the manifestation of their gain-of-function (GOF) properties. In cancer cells mutant p53 proteins are stabilized by the interaction with Heat shock protein 90 (Hsp90) chaperones. This binding results in the formation of complexes protecting mutant p53 from E3 ligase MDM2 ubiquitination. The increase in matrix stiffness is a common feature in solid tumors, and in this context the small GTPase RhoA (Ras Homolog Family Member A) plays an important role, by transducing the mechanical stimulus from the extracellular environment. RhoA-mediated mechanotransduction has been shown to promote the accumulation of mutant p53. In particular, RhoA signaling activation has been shown to promote the binding of Hsp90 to mutant p53, resulting in its stabilization. The RhoA mediated mechanosignaling in turn requires the mevalonate pathway. Indeed, in addition to cholesterol biosynthesis, this pathway also provides geranylgeranyl pyrophosphate (GGPP) that is required for cellular membrane anchoring, and activation of RhoA (Figure 3) [118]. Thus, p53 mutants promote the mevalonate pathway [103], whose activation in turn induces the accumulation of mutant p53 protein in cancer cells.

## 7. Tumor Dependency to Mutant p53

Within tumors, cancer cells are constantly exposed to several stresses such as hypoxia, starvation, and exposure to anticancer drugs. To survive, cancer cells can develop adaptation to stress conditions, such as the eventual mutation of p53 at a very late stage. Indeed, as discussed in the above sections, p53 mutations cause loss of tumor suppressive functions as well as gain of new pro-tumorigenic activity, that allows cancer cells to adapt to the challenging conditions typically present in the tumor. Thus, it is not surprising that tumor cells can develop a dependency to mutant p53 expression. The constitutive depletion of mutant p53 results in a decrease in tumor growth, invasion, and angiogenesis in nude mice [119]. In addition, mutant p53 depletion in breast cancer cells in 3D culture lead to a phenotypic reversion from a disordered and invasive morphology to more physiological, differentiated structures. A further study using a conditional mutant *p53* mouse model expressing an inactivable R248Q mutation, showed that mutant *p53* ablation decreases tumor growth extending animal survival [120]. These evidences have clearly demonstrated that most of the cancer cells expressing mutant p53 require its expression to survive, or at least maintain their tumorigenic capabilities. In a recent in vivo study of a mouse model of colorectal cancer (CRC), mutant p53 R248Q was shown to interact with phospho- Signal Transducer And Activator Of Transcription 3 (p-STAT3), to block its interaction with the tyrosine phosphatase SHP2 (Protein Tyrosine Phosphatase Non-Receptor Type 11). Consequently, the binding of mutant p53 R248Q to pSTAT3 prevents its dephosphorylation, leading to the hyperactivation of STAT3 signaling pathway that in turn drives cancer progression, by inducing the expression of target genes such as *CCND1* (Cyclin D1), *CCNB1* (Cyclin B1), and *MYC* (MYC Proto-Oncogene,) (Figure 2A). The genetic ablation of mutant p53 R248Q reduces tumor growth and invasion [121]. All together these data indicate that tumors display a dependency to mutant p53 expression to sustain their growth, posing mutant p53 as an attractive target for cancer therapy. Identification of the basis of control of mutant p53 expression and protein stability therefore represents a priority in the field, to develop strategies to target mutant p53 expressing tumors.

## 8. Targeting Mutant p53 for Therapy

To date, several strategies are being explored to target mutant p53 for cancer therapy. One approach uses small molecular compounds to directly target mutant p53, in order to induce its degradation or the restoration of its transcriptional tumor-suppressive activity. A second approach exploits tumor addiction to mutant p53 GOF targeting pathways, induced by gain-of-function p53 mutants. Another promising strategy aims to promote p53 function by targeting its antagonists such as the E3 ligase, Mdm2.

### 8.1. Therapies to Restore Wild Type p53 Functions

As expected, re-expression of wild type p53 in p53-null or p53 mutant tumors is sufficient to induce tumor regression [122,123]. Therefore, significant efforts have been focused to develop strategies based on small molecules that could reactivate wild type p53 functions in tumor cells carrying mutant p53 proteins. The main goal of these mutant p53 targeting compounds is to promote a conformational change in mutant p53 folding, in order to restore the physiological transcriptional activity of p53 and induce it anti-tumor activity. As one of the first developed compounds, PRIMA-1 can induce mutant p53 proteins to refold into a wild type conformation, restoring wild type p53-like transcriptional activity, and inducing the expression of *PUMA*, *NOXA*, and *BAX* target genes [124,125]. The methylated analogue of PRIMA-1, PRIMA-1^Met^ (APR-246), is more potent and less toxic [126]. At the chemical level, PRIMA-1 is converted inside cells into the active form methylene quinuclidinone (MQ). The interaction of this metabolite with cysteine residues of mutant p53 protein causes its proper refolding to wild type p53 [127]. PRIMA-1 is a prototype compound for this activity; however, the specificity of its effects is still being discussed, as part of its cytotoxic effect seems to be associated with alterations to cellular antioxidant machinery. To date, PRIMA-1 is undergoing phase 3 clinical trials, and represents a promising alternative therapeutic strategy for cancer patients.

### 8.2. Therapies to Induce Mutant p53 Degradation

Detection of p53 protein expression in tumor tissue is a read-out of p53 mutant status. In cancers, mutant p53 is indeed much more stable than wild type p53, and positive staining is generally observed in primary material as well as in cell lines. Mutant p53 stability has been ascribed to its interaction with the Histone Deacetylase 6/ Heat Shock 90kD Protein 1 (HDAC6/Hsp90) chaperone complex that stabilizes mutant p53, preventing its degradation mediated by MDM2 E3 ubiquitin ligase [128,129]. Therefore, compounds able to disrupt this HDAC6/HSP90 complex are being developed in order to promote mutant p53 degradation. These compounds, including both Hsp90 inhibitors (17-AAG and ganetespib) and HDAC inhibitors (such as SAHA or vorinostat), are able to promote proteasome-dependent degradation of mutant p53 [120,130]. Another class of compounds able to induce degradation of mutant p53 proteins are statins. These compounds preferentially induce degradation of conformational p53 mutants. Mechanistically, statins inhibit the mevalonate-5-phosphate pathway and induce E3 Ubiquitin-Protein Ligase CHIP -mediated degradation of mutant p53, by impairing the interaction of mutant p53 with DNAJA1 (DnaJ Heat Shock Protein Family Member A1), an Hsp40 isoform, similarly to Hsp90s involved in mutant p53 protection [131]. As mentioned above, statins also degrade mutant p53 proteins by interfering with the mevalonate-geranylgeranyl-pyrophosphate-RhoA mechanosignaling pathway, which controls Hsp90-dependent p53 mutant stabilization [118]. Overall, bi-directional interactions between mutant p53 and the mevalonic pathway could be a promising therapeutic target.

### 8.3. Targeting Mutant p53 GOF

The novel oncogenic functions acquired by mutant p53 proteins contribute to tumor development and progression. For example, p53 mutants can activate cell migratory pathways able to stimulate migration, invasion, and metastasis. Therefore, the inhibition of the downstream pathways triggered by mutant p53 GOF represents an alternative strategy for effective treatment of p53-mutant cancers. For example, in a mouse model of pancreatic ductal adenocarcinoma (PDAC) mutant p53 promoted invasion and metastasis, by enhancing platelet-derived growth factor receptor beta (PDGFRβ) signaling. Interestingly, treatment of p53-mutant pancreatic cancers with imatinib, an inhibitor of PDGFRβ signaling, is effectively able to impair cell invasion and metastasis [91]. Another pathway that could be targeted for the treatment of p53-mutant cancers is the RhoA/ROCK pathway. Mutant p53 R172H has been shown to increased Ras Homolog Family Member A /Rho Associated Coiled-Coil Containing Protein Kinase (RhoA/ROCK) signaling that in turn promotes glucose transporter GLUT1 (Glucose Transporter Type 1) translocation to the plasma membrane (Figure 2B). This results in an increased glucose uptake that promotes glycolysis, and ultimately tumorigenesis. An in vivo experiment showed that Dasatanib treatment inhibited the activity of RhoA and impaired the invasive potential of p53 R172H cells [132].

### 8.4. Targeting p53 in Wild Type p53 Tumours: MDM2 Inhibition

MDM2 is a E3 ubiquitin ligase that controls cellular levels of p53. In unstressed conditions, MDM2 maintains low levels of p53 by mediating its proteasomal-dependent degradation. In response to stress, both MDM2 and p53 undergo post-translational modifications that impair their interaction, allowing p53 to accumulate and exert its tumor-suppressive functions. Interestingly, the *mdm2* gene is amplified in more than 17% of tumors resulting in p53 inactivation. Thus, a current therapeutic strategy is based on the use of drugs able to block MDM2, which include MDM2 antagonists (such as Nutlin-3) and inhibitors of MDM2-p53 interaction (MI-219 and MI-319), in p53 wild type tumors [133,134].

## 9. Conclusions

Mutations in p53 have far reaching consequences for the biology of the cancer cells, especially when associated with the expression of neomorphic p53 proteins. GOF p53 mutants can help malignant cells to survive and adapt to stresses, such as hypoxia, nutrient deprivation, etc. Thus, tumor cells obtain selective advantages by maintaining the mutant forms of the protein, beyond the loss of wt p53 function. The signaling triggered by GOF proteins might underlie the cancers addiction to mutant p53, and this opens potential therapeutic strategies to target aggressive late stage cancers. While a better understanding of GOF mechanisms is still needed [129,135], a pragmatic effort should also be invested in, developing approaches to promote degradation of mutant p53 to treat lethal cancers.

## Figures and Tables

**Table 1:** Frequency of six hot spot p53 mutations in several human cancers. The frequenty of both somatic and germline p53 mutation is shown.

**TUMOR TYPES**	**SOMATIC MUTATIONS FREQUENCY (%)**					
	**R175H**	**R273H**	**R248Q**	**R248W**	**G245S**	**R273C**
BILIARY TRACT	10.96	6.85	2.74	4.11		2.74
BLADDER	2.51	1.39	3.50	1.65	1.19	0.99
BONES	4.20	3.36	4.20	3.77	1.26	5.88
BRAIN	5.80	4.34	4.07	1.26	2.01	10.63
BREAST	4.52	3.30	3.72	3.09	1.39	1.22
CERVIX UTERI	4.27	2.56	5.13	2.85	1.71	5.13
COLON	8.56	5.68	4.98	1.71	4.02	4.45
COLORECTUM, NOS	10.91	5.68	5.74	4.54	3.86	2.39
CORPUS UTERI	2.76	3.23	3.69		0.92	2.30
ESOPHAGUS	5.07	2.45	2.61	3.09	1.39	1.39
GALLBLADDER	4.55	0.91	2.73			
GUM	4.94	1.23	1.23	2.47	1.23	3.70
HEAD & NECK, NOS	2.56	1.50	2.41	1.95	0.75	1.05
HEMATOP. SYSTEM	4.71	2.68	8.89	1.61	1.71	1.71
HYPOPHARYNX	2.73	2.19	2.19	1.64	1.64	0.55
KIDNEY	3.40	1.36	3.40	2.72	1.36	1.36
LARYNX	1.37	2.06	1.83	1.60	0.23	1.37
LIVER	1.09	0.50	1.17	0.58	0.83	2.09
LUNG	1.21	1.77	1.21	1.54	0.33	0.88
LYMPH NODES	3.28	2.49	6.29	2.36	1.18	3.01
MOUTH (floor)	3.19	3.19	3.19			
MOUTH (other)	3.64	0.87	3.20	1.16	1.31	1.75
NASAL CAVITY	1.05	2.11	0.53	1.58	1.05	0.53
NASOPHARYNX	4.84	24.19	3.23	1.61		
OTHER FEMALE GEN. ORG.	16.00	4.00	4.00	4.00	4.00	4.00
OTHER RESPIR. SYST.	9.09	13.64	4.55	4.55		
OVARY	4.64	4.08	2.34	2.52	1.43	2.30
PANCREAS	3.67	6.52	2.24	2.65	1.02	3.67
PENIS	14.29	28.57				7.14
PERITONEUM	2.17	4.35	2.17			
PROSTATE	2.41	2.14	1.88	1.34	1.07	4.83
RECTOSIGM. JUNCT.	12.50	2.50	5.00	5.00		7.50
RECTUM	10.27	4.92	4.78		4.78	4.20
SINUSES	4.57	1.83	2.74	2.28	0.46	0.91
SKIN	0.57	0.57	1.81	4.37	1.05	0.57
SOFT TISSUES	1.69	2.66	1.94	2.18	2.42	0.97
STOMACH	6.84	3.17	3.58	3.17	3.17	2.76
TONGUE (other)	1.44	1.91	2.87	1.44	1.44	1.44
URINARY TRACT, NOS	20.00	0.58	1.16			0.58
UTERUS	5.48	2.74		4.11		6.85
VULVA	2.78	2.78	6.48	0.93	4.63	3.70
**TUMOR TYPES**	**GERMLINE MUTATIONS FREQUENCY (%)**					
	**R175H**	**R273H**	**R248Q**	**R248W**	**G245S**	**R273C**
ADRENAL GLAND	6.96	3.09	4.0	7.21	1.3	1.64
BONES	8.7	14.43	11.0	6.31	3.9	3.28
BRAIN	14.78	8.25	11.0	24.32		9.84
BREAST	29.57	24.74	29.0	29.73		27.87
HEMATOP. SYSTEM	4.35	4.12	2.0		6.49	6.56
LUNG	0.87	4.12	3.0	2.7	5.19	6.56
SKIN	2.61	2.06	1.0	2.7	6.49	8.2
SOFT TISSUES	12.17	18.56	16.0		11.69	6.56
STOMACH	3.48	4.12		6.31	1.3	1.64
THYROID	0.87	1.03	2.0	3.6		3.28

## References

[1] Dolgin E. (2017). The most popular genes in the human genome. Nature.

[2] Kaiser A.M., Attardi L.D. (2018). Deconstructing networks of p53-mediated tumor suppression in vivo. Cell Death Differ..

[3] Bieging K.T., Mello S.S., Attardi L.D. (2014). Unravelling mechanisms of p53-mediated tumour suppression. Nat. Rev. Cancer.

[4] Aubrey B.J., Kelly G.L., Janic A., Herold M.J., Strasser A. (2018). How does p53 induce apoptosis and how does this relate to p53-mediated tumour suppression?. Cell Death Differ..

[5] Kubbutat M.H., Jones S.N., Vousden K.H. (1997). Regulation of p53 stability by Mdm2. Nature.

[6] Honda R., Tanaka H., Yasuda H. (1997). Oncoprotein MDM2 is a ubiquitin ligase E3 for tumor suppressor p53. Febs. Lett..

[7] Haupt Y., Maya R., Kazaz A., Oren M. (1997). Mdm2 promotes the rapid degradation of p53. Nature.

[8] Wu D., Prives C. (2018). Relevance of the p53-MDM2 axis to aging. Cell Death Differ..

[9] Chen J., Marechal V., Levine A.J. (1993). Mapping of the p53 and mdm-2 interaction domains. Mol. Cell Biol..

[10] Barak Y., Juven T., Haffner R., Oren M. (1993). mdm2 expression is induced by wild type p53 activity. EMBO J..

[11] Wu L., Levine A.J. (1997). Differential regulation of the p21/WAF-1 and mdm2 genes after high-dose UV irradiation: p53-dependent and p53-independent regulation of the mdm2 gene. Mol. Med..

[12] Arena G., Riscal R., Linares L.K., Le Cam L. (2018). MDM2 controls gene expression independently of p53 in both normal and cancer cells. Cell Death Differ..

[13] Shieh S.Y., Ikeda M., Taya Y., Prives C. (1997). DNA damage-induced phosphorylation of p53 alleviates inhibition by MDM2. Cell.

[14] Williams A.B., Schumacher B. (2016). p53 in the DNA-Damage-Repair Process. Cold Spring Harb. Perspect. Med..

[15] Lim Y., De Bellis D., Dorstyn L., Kumar S. (2018). p53 accumulation following cytokinesis failure in the absence of caspase-2. Cell Death Differ..

[16] Sankunny M., Eng C. (2018). KLLN-mediated DNA damage-induced apoptosis is associated with regulation of p53 phosphorylation and acetylation in breast cancer cells. Cell Death Discov..

[17] Hunger A., Medrano R.F., Zanatta D.B., Del Valle P.R., Merkel C.A., Salles T.A., Ferrari D.G., Furuya T.K., Bustos S.O., de Freitas Saito R. (2017). Reestablishment of p53/Arf and interferon-beta pathways mediated by a novel adenoviral vector potentiates antiviral response and immunogenic cell death. Cell Death Discov..

[18] Chen J. (2016). The Cell-Cycle Arrest and Apoptotic Functions of p53 in Tumor Initiation and Progression. Cold Spring Harb. Perspect. Med..

[19] Levine A.J., Oren M. (2009). The first 30 years of p53: Growing ever more complex. Nat. Rev. Cancer.

[20] Sullivan K.D., Galbraith M.D., Andrysik Z., Espinosa J.M. (2018). Mechanisms of transcriptional regulation by p53. Cell Death Differ..

[21] El-Deiry W.S., Tokino T., Velculescu V.E., Levy D.B., Parsons R., Trent J.M., Lin D., Mercer W.E., Kinzler K.W., Vogelstein B. (1993). WAF1, a potential mediator of p53 tumor suppression. Cell.

[22] Harper J.W., Adami G.R., Wei N., Keyomarsi K., Elledge S.J. (1993). The p21 Cdk-interacting protein Cip1 is a potent inhibitor of G1 cyclin-dependent kinases. Cell.

[23] Engeland K. (2018). Cell cycle arrest through indirect transcriptional repression by p53: I have a DREAM. Cell Death Differ..

[24] Vazquez A., Bond E.E., Levine A.J., Bond G.L. (2008). The genetics of the p53 pathway, apoptosis and cancer therapy. Nat. Rev. Drug. Discov..

[25] Riley T., Sontag E., Chen P., Levine A. (2008). Transcriptional control of human p53-regulated genes. Nat. Rev. Mol. Cell Biol..

[26] Lopez I., Tournillon A.S., Prado Martins R., Karakostis K., Malbert-Colas L., Nylander K., Fahraeus R. (2017). p53-mediated suppression of BiP triggers BIK-induced apoptosis during prolonged endoplasmic reticulum stress. Cell Death Differ..

[27] Janic A., Valente L.J., Wakefield M.J., Di Stefano L., Milla L., Wilcox S., Yang H., Tai L., Vandenberg C.J., Kueh A.J. (2018). DNA repair processes are critical mediators of p53-dependent tumor suppression. Nat. Med..

[28] Adimoolam S., Ford J.M. (2002). p53 and DNA damage-inducible expression of the xeroderma pigmentosum group C gene. Proc. Natl. Acad. Sci. USA.

[29] Chatterjee A., Mambo E., Osada M., Upadhyay S., Sidransky D. (2006). The effect of p53-RNAi and p53 knockout on human 8-oxoguanine DNA glycosylase (hOgg1) activity. FASEB J..

[30] Oka S., Leon J., Tsuchimoto D., Sakumi K., Nakabeppu Y. (2014). MUTYH, an adenine DNA glycosylase, mediates p53 tumor suppression via PARP-dependent cell death. Oncogenesis.

[31] Zurer I., Hofseth L.J., Cohen Y., Xu-Welliver M., Hussain S.P., Harris C.C., Rotter V. (2004). The role of p53 in base excision repair following genotoxic stress. Carcinogenesis.

[32] Hamann I., Konig C., Richter C., Jahnke G., Hartwig A. (2012). Impact of cadmium on hOGG1 and APE1 as a function of the cellular p53 status. Mutat. Res..

[33] Scherer S.J., Maier S.M., Seifert M., Hanselmann R.G., Zang K.D., Muller-Hermelink H.K., Angel P., Welter C., Schartl M. (2000). p53 and c-Jun functionally synergize in the regulation of the DNA repair gene hMSH2 in response to UV. J. Biol. Chem..

[34] Akyuz N., Boehden G.S., Susse S., Rimek A., Preuss U., Scheidtmann K.H., Wiesmuller L. (2002). DNA substrate dependence of p53-mediated regulation of double-strand break repair. Mol. Cell Biol..

[35] Lin Y., Waldman B.C., Waldman A.S. (2003). Suppression of high-fidelity double-strand break repair in mammalian chromosomes by pifithrin-alpha, a chemical inhibitor of p53. DNA Repair (Amst).

[36] Hine C.M., Li H., Xie L., Mao Z., Seluanov A., Gorbunova V. (2014). Regulation of Rad51 promoter. Cell Cycle.

[37] Moll U.M., Wolff S., Speidel D., Deppert W. (2005). Transcription-independent pro-apoptotic functions of p53. Curr. Opin. Cell Biol..

[38] Wang X.W., Yeh H., Schaeffer L., Roy R., Moncollin V., Egly J.M., Wang Z., Freidberg E.C., Evans M.K., Taffe B.G. (1995). p53 modulation of TFIIH-associated nucleotide excision repair activity. Nat. Genet..

[39] Leveillard T., Andera L., Bissonnette N., Schaeffer L., Bracco L., Egly J.M., Wasylyk B. (1996). Functional interactions between p53 and the TFIIH complex are affected by tumour-associated mutations. EMBO J..

[40] Seo Y.R., Fishel M.L., Amundson S., Kelley M.R., Smith M.L. (2002). Implication of p53 in base excision DNA repair: In vivo evidence. Oncogene.

[41] Linke S.P., Sengupta S., Khabie N., Jeffries B.A., Buchhop S., Miska S., Henning W., Pedeux R., Wang X.W., Hofseth L.J. (2003). p53 interacts with hRAD51 and hRAD54, and directly modulates homologous recombination. Cancer Res..

[42] Brady C.A., Attardi L.D. (2010). p53 at a glance. J. Cell Sci..

[43] Ranjan A., Iwakuma T. (2018). Emerging Non-Canonical Functions and Regulation of p53. Int. J. Mol. Sci..

[44] Mello S.S., Attardi L.D. (2018). Deciphering p53 signaling in tumor suppression. Curr. Opin. Cell Biol..

[45] Charni M., Aloni-Grinstein R., Molchadsky A., Rotter V. (2017). p53 on the crossroad between regeneration and cancer. Cell Death Differ..

[46] Huun J., Lonning P.E., Knappskog S. (2017). Effects of concomitant inactivation of p53 and pRb on response to doxorubicin treatment in breast cancer cell lines. Cell Death Discov..

[47] Olivier M., Hollstein M., Hainaut P. (2010). TP53 mutations in human cancers: Origins, consequences, and clinical use. Cold Spring Harb. Perspect. Biol..

[48] Malkin D., Li F.P., Strong L.C., Fraumeni J.F., Nelson C.E., Kim D.H., Kassel J., Gryka M.A., Bischoff F.Z., Tainsky M.A. (1990). Germ line p53 mutations in a familial syndrome of breast cancer, sarcomas, and other neoplasms. Science.

[49] Donehower L.A., Harvey M., Slagle B.L., McArthur M.J., Montgomery C.A., Butel J.S., Bradley A. (1992). Mice deficient for p53 are developmentally normal but susceptible to spontaneous tumours. Nature.

[50] Olivier M., Hussain S.P., Caron de Fromentel C., Hainaut P., Harris C.C. (2004). TP53 mutation spectra and load: A tool for generating hypotheses on the etiology of cancer. IARC Sci. Publ..

[51] Pfister N.T., Prives C. (2017). Transcriptional Regulation by Wild-Type and Cancer-Related Mutant Forms of p53. Cold Spring Harb. Perspect. Med..

[52] Yue X., Zhao Y., Xu Y., Zheng M., Feng Z., Hu W. (2017). Mutant p53 in Cancer: Accumulation, Gain-of-Function, and Therapy. J. Mol. Biol..

[53] Bargonetti J., Friedman P.N., Kern S.E., Vogelstein B., Prives C. (1991). Wild-type but not mutant p53 immunopurified proteins bind to sequences adjacent to the SV40 origin of replication. Cell.

[54] Kern S.E., Kinzler K.W., Bruskin A., Jarosz D., Friedman P., Prives C., Vogelstein B. (1991). Identification of p53 as a sequence-specific DNA-binding protein. Science.

[55] Milner J., Medcalf E.A. (1991). Cotranslation of activated mutant p53 with wild type drives the wild-type p53 protein into the mutant conformation. Cell.

[56] Milner J., Medcalf E.A., Cook A.C. (1991). Tumor suppressor p53: Analysis of wild-type and mutant p53 complexes. Mol. Cell Biol..

[57] Willis A., Jung E.J., Wakefield T., Chen X. (2004). Mutant p53 exerts a dominant negative effect by preventing wild-type p53 from binding to the promoter of its target genes. Oncogene.

[58] Baker S.J., Preisinger A.C., Jessup J.M., Paraskeva C., Markowitz S., Willson J.K., Hamilton S., Vogelstein B. (1990). p53 gene mutations occur in combination with 17p allelic deletions as late events in colorectal tumorigenesis. Cancer Res..

[59] Alexandrova E.M., Mirza S.A., Xu S., Schulz-Heddergott R., Marchenko N.D., Moll U.M. (2017). p53 loss-of-heterozygosity is a necessary prerequisite for mutant p53 stabilization and gain-of-function in vivo. Cell Death Dis..

[60] Lang G.A., Iwakuma T., Suh Y.A., Liu G., Rao V.A., Parant J.M., Valentin-Vega Y.A., Terzian T., Caldwell L.C., Strong L.C. (2004). Gain of function of a p53 hot spot mutation in a mouse model of Li-Fraumeni syndrome. Cell.

[61] Olive K.P., Tuveson D.A., Ruhe Z.C., Yin B., Willis N.A., Bronson R.T., Crowley D., Jacks T. (2004). Mutant p53 gain of function in two mouse models of Li-Fraumeni syndrome. Cell.

[62] Bougeard G., Sesboue R., Baert-Desurmont S., Vasseur S., Martin C., Tinat J., Brugieres L., Chompret A., de Paillerets B.B., Stoppa-Lyonnet D. (2008). Molecular basis of the Li-Fraumeni syndrome: An update from the French LFS families. J. Med. Genet..

[63] Hanel W., Marchenko N., Xu S., Yu S.X., Weng W., Moll U. (2013). Two hot spot mutant p53 mouse models display differential gain of function in tumorigenesis. Cell Death Differ..

[64] Xu J., Qian J., Hu Y., Wang J., Zhou X., Chen H., Fang J.Y. (2014). Heterogeneity of Li-Fraumeni syndrome links to unequal gain-of-function effects of p53 mutations. Sci. Rep..

[65] Fearon E.R., Vogelstein B. (1990). A genetic model for colorectal tumorigenesis. Cell.

[66] Hruban R.H., Goggins M., Parsons J., Kern S.E. (2000). Progression model for pancreatic cancer. Clin. Cancer Res..

[67] Olivier M., Langerod A., Carrieri P., Bergh J., Klaar S., Eyfjord J., Theillet C., Rodriguez C., Lidereau R., Bieche I. (2006). The clinical value of somatic TP53 gene mutations in 1,794 patients with breast cancer. Clin. Cancer Res..

[68] Yachida S., Iacobuzio-Donahue C.A. (2013). Evolution and dynamics of pancreatic cancer progression. Oncogene.

[69] Kim M.P., Lozano G. (2018). Mutant p53 partners in crime. Cell Death Differ..

[70] Aas T., Borresen A.L., Geisler S., Smith-Sorensen B., Johnsen H., Varhaug J.E., Akslen L.A., Lonning P.E. (1996). Specific P53 mutations are associated with de novo resistance to doxorubicin in breast cancer patients. Nat. Med..

[71] Shelling A.N. (1997). Role of p53 in drug resistance in ovarian cancer. Lancet.

[72] Horio Y., Takahashi T., Kuroishi T., Hibi K., Suyama M., Niimi T., Shimokata K., Yamakawa K., Nakamura Y., Ueda R. (1993). Prognostic significance of p53 mutations and 3p deletions in primary resected non-small cell lung cancer. Cancer Res..

[73] Hamada M., Fujiwara T., Hizuta A., Gochi A., Naomoto Y., Takakura N., Takahashi K., Roth J.A., Tanaka N., Orita K. (1996). The p53 gene is a potent determinant of chemosensitivity and radiosensitivity in gastric and colorectal cancers. J. Cancer Res. Clin. Oncol..

[74] Wijdeven R.H., Pang B., Assaraf Y.G., Neefjes J. (2016). Old drugs, novel ways out: Drug resistance toward cytotoxic chemotherapeutics. Drug Resist. Updates.

[75] Chin K.V., Ueda K., Pastan I., Gottesman M.M. (1992). Modulation of activity of the promoter of the human MDR1 gene by Ras and p53. Science.

[76] Song H., Hollstein M., Xu Y. (2007). p53 gain-of-function cancer mutants induce genetic instability by inactivating ATM. Nat. Cell Biol..

[77] Polotskaia A., Xiao G., Reynoso K., Martin C., Qiu W.G., Hendrickson R.C., Bargonetti J. (2015). Proteome-wide analysis of mutant p53 targets in breast cancer identifies new levels of gain-of-function that influence PARP, PCNA, and MCM4. Proc. Natl. Acad. Sci. USA.

[78] Kuerbitz S.J., Plunkett B.S., Walsh W.V., Kastan M.B. (1992). Wild-type p53 is a cell cycle checkpoint determinant following irradiation. Proc. Natl. Acad. Sci. USA.

[79] Lowe S.W., Bodis S., McClatchey A., Remington L., Ruley H.E., Fisher D.E., Housman D.E., Jacks T. (1994). p53 status and the efficacy of cancer therapy in vivo. Science.

[80] Lee J.M., Bernstein A. (1993). p53 mutations increase resistance to ionizing radiation. Proc. Natl. Acad. Sci. USA.

[81] Bergh J., Norberg T., Sjogren S., Lindgren A., Holmberg L. (1995). Complete sequencing of the p53 gene provides prognostic information in breast cancer patients, particularly in relation to adjuvant systemic therapy and radiotherapy. Nat. Med..

[82] Belyi V.A., Levine A.J. (2009). One billion years of p53/p63/p73 evolution. Proc. Natl. Acad. Sci. USA.

[83] Belyi V.A., Ak P., Markert E., Wang H., Hu W., Puzio-Kuter A., Levine A.J. (2010). The origins and evolution of the p53 family of genes. Cold Spring Harb. Perspect. Biol..

[84] Kehrloesser S., Osterburg C., Tuppi M., Schafer B., Vousden K.H., Dotsch V. (2016). Intrinsic aggregation propensity of the p63 and p73 TI domains correlates with p53R175H interaction and suggests further significance of aggregation events in the p53 family. Cell Death Differ..

[85] Gaiddon C., Lokshin M., Ahn J., Zhang T., Prives C. (2001). A subset of tumor-derived mutant forms of p53 down-regulate p63 and p73 through a direct interaction with the p53 core domain. Mol. Cell Biol..

[86] Ghosh S., Salot S., Sengupta S., Navalkar A., Ghosh D., Jacob R., Das S., Kumar R., Jha N.N., Sahay S. (2017). p53 amyloid formation leading to its loss of function: Implications in cancer pathogenesis. Cell Death Differ..

[87] Rada M., Barlev N., Macip S. (2018). BTK modulates p73 activity to induce apoptosis independently of p53. Cell Death Discov..

[88] Adorno M., Cordenonsi M., Montagner M., Dupont S., Wong C., Hann B., Solari A., Bobisse S., Rondina M.B., Guzzardo V. (2009). A Mutant-p53/Smad complex opposes p63 to empower TGFbeta-induced metastasis. Cell.

[89] Miyazaki M., Otomo R., Matsushima-Hibiya Y., Suzuki H., Nakajima A., Abe N., Tomiyama A., Ichimura K., Matsuda K., Watanabe T. (2018). The p53 activator overcomes resistance to ALK inhibitors by regulating p53-target selectivity in ALK-driven neuroblastomas. Cell Death Discov..

[90] Muller P.A., Caswell P.T., Doyle B., Iwanicki M.P., Tan E.H., Karim S., Lukashchuk N., Gillespie D.A., Ludwig R.L., Gosselin P. (2009). Mutant p53 drives invasion by promoting integrin recycling. Cell.

[91] Weissmueller S., Manchado E., Saborowski M., Morris J.P.t., Wagenblast E., Davis C.A., Moon S.H., Pfister N.T., Tschaharganeh D.F., Kitzing T. (2014). Mutant p53 drives pancreatic cancer metastasis through cell-autonomous PDGF receptor beta signaling. Cell.

[92] Zheng S., Xin L., Liang A., Fu Y. (2013). Cancer stem cell hypothesis: A brief summary and two proposals. Cytotechnology.

[93] Valent P., Bonnet D., De Maria R., Lapidot T., Copland M., Melo J.V., Chomienne C., Ishikawa F., Schuringa J.J., Stassi G. (2012). Cancer stem cell definitions and terminology: The devil is in the details. Nat. Rev. Cancer.

[94] Levine A.J., Berger S.L. (2017). The interplay between epigenetic changes and the p53 protein in stem cells. Genes Dev..

[95] Koifman G., Shetzer Y., Eizenberger S., Solomon H., Rotkopf R., Molchadsky A., Lonetto G., Goldfinger N., Rotter V. (2018). A Mutant p53-Dependent Embryonic Stem Cell Gene Signature Is Associated with Augmented Tumorigenesis of Stem Cells. Cancer Res..

[96] Petitjean A., Mathe E., Kato S., Ishioka C., Tavtigian S.V., Hainaut P., Olivier M. (2007). Impact of mutant p53 functional properties on TP53 mutation patterns and tumor phenotype: Lessons from recent developments in the IARC TP53 database. Hum. Mutat..

[97] Li N., Yang R., Zhang W., Dorfman H., Rao P., Gorlick R. (2009). Genetically transforming human mesenchymal stem cells to sarcomas: Changes in cellular phenotype and multilineage differentiation potential. Cancer.

[98] Mohseny A.B., Hogendoorn P.C. (2011). Concise review: Mesenchymal tumors: When stem cells go mad. Stem Cells.

[99] Shetzer Y., Kagan S., Koifman G., Sarig R., Kogan-Sakin I., Charni M., Kaufman T., Zapatka M., Molchadsky A., Rivlin N. (2014). The onset of p53 loss of heterozygosity is differentially induced in various stem cell types and may involve the loss of either allele. Cell Death Differ..

[100] Di Fiore R., Marcatti M., Drago-Ferrante R., D’Anneo A., Giuliano M., Carlisi D., De Blasio A., Querques F., Pastore L., Tesoriere G. (2014). Mutant p53 gain of function can be at the root of dedifferentiation of human osteosarcoma MG63 cells into 3AB-OS cancer stem cells. Bone.

[101] Loizou E., Banito A., Livshits G., Ho Y.J., Koche R.P., Sanchez-Rivera F.J., Mayle A., Chen C.C., Kinalis S., Bagger F.O. (2019). A Gain-of-Function p53-Mutant Oncogene Promotes Cell Fate Plasticity and Myeloid Leukemia through the Pluripotency Factor FOXH1. Cancer Discov..

[102] Eberle D., Hegarty B., Bossard P., Ferre P., Foufelle F. (2004). SREBP transcription factors: Master regulators of lipid homeostasis. Biochimie.

[103] Freed-Pastor W.A., Mizuno H., Zhao X., Langerod A., Moon S.H., Rodriguez-Barrueco R., Barsotti A., Chicas A., Li W., Polotskaia A. (2012). Mutant p53 disrupts mammary tissue architecture via the mevalonate pathway. Cell.

[104] Clendening J.W., Pandyra A., Boutros P.C., El Ghamrasni S., Khosravi F., Trentin G.A., Martirosyan A., Hakem A., Hakem R., Jurisica I. (2010). Dysregulation of the mevalonate pathway promotes transformation. Proc. Natl. Acad. Sci. USA.

[105] Parrales A., Thoenen E., Iwakuma T. (2018). The interplay between mutant p53 and the mevalonate pathway. Cell Death Differ..

[106] Sorrentino G., Mantovani F., Del Sal G. (2018). The stiff RhoAd from mevalonate to mutant p53. Cell Death Differ..

[107] Moon S.H., Huang C.H., Houlihan S.L., Regunath K., Freed-Pastor W.A., Morris J.P.t., Tschaharganeh D.F., Kastenhuber E.R., Barsotti A.M., Culp-Hill R. (2019). p53 Represses the Mevalonate Pathway to Mediate Tumor Suppression. Cell.

[108] Garcia D., Shaw R.J. (2017). AMPK: Mechanisms of Cellular Energy Sensing and Restoration of Metabolic Balance. Mol. Cell.

[109] Zhou G., Wang J., Zhao M., Xie T.X., Tanaka N., Sano D., Patel A.A., Ward A.M., Sandulache V.C., Jasser S.A. (2014). Gain-of-function mutant p53 promotes cell growth and cancer cell metabolism via inhibition of AMPK activation. Mol. Cell.

[110] Xu R., Garcia-Barros M., Wen S., Li F., Lin C.L., Hannun Y.A., Obeid L.M., Mao C. (2018). Tumor suppressor p53 links ceramide metabolism to DNA damage response through alkaline ceramidase 2. Cell Death Differ..

[111] Muz B., de la Puente P., Azab F., Azab A.K. (2015). The role of hypoxia in cancer progression, angiogenesis, metastasis, and resistance to therapy. Hypoxia (Auckl).

[112] Li L., Yang H., Li Y., Li X.D., Zeng T.T., Lin S.X., Zhu Y.H., Guan X.Y. (2018). Hypoxia restrains the expression of complement component 9 in tumor-associated macrophages promoting non-small cell lung cancer progression. Cell Death Discov..

[113] Narendran A., Ganjavi H., Morson N., Connor A., Barlow J.W., Keystone E., Malkin D., Freedman M.H. (2003). Mutant p53 in bone marrow stromal cells increases VEGF expression and supports leukemia cell growth. Exp. Hematol..

[114] Fontemaggi G., Dell’Orso S., Trisciuoglio D., Shay T., Melucci E., Fazi F., Terrenato I., Mottolese M., Muti P., Domany E. (2009). The execution of the transcriptional axis mutant p53, E2F1 and ID4 promotes tumor neo-angiogenesis. Nat. Struct. Mol. Biol..

[115] Pfister N.T., Fomin V., Regunath K., Zhou J.Y., Zhou W., Silwal-Pandit L., Freed-Pastor W.A., Laptenko O., Neo S.P., Bargonetti J. (2015). Mutant p53 cooperates with the SWI/SNF chromatin remodeling complex to regulate VEGFR2 in breast cancer cells. Genes Dev..

[116] Amelio I., Mancini M., Petrova V., Cairns R.A., Vikhreva P., Nicolai S., Marini A., Antonov A.A., Le Quesne J., Baena Acevedo J.D. (2018). p53 mutants cooperate with HIF-1 in transcriptional regulation of extracellular matrix components to promote tumor progression. Proc. Natl. Acad. Sci. USA.

[117] Gouirand V., Vasseur S. (2018). Fountain of youth of pancreatic cancer cells: The extracellular matrix. Cell Death Discov..

[118] Ingallina E., Sorrentino G., Bertolio R., Lisek K., Zannini A., Azzolin L., Severino L.U., Scaini D., Mano M., Mantovani F. (2018). Mechanical cues control mutant p53 stability through a mevalonate-RhoA axis. Nat. Cell Biol..

[119] Bossi G., Marampon F., Maor-Aloni R., Zani B., Rotter V., Oren M., Strano S., Blandino G., Sacchi A. (2008). Conditional RNA interference in vivo to study mutant p53 oncogenic gain of function on tumor malignancy. Cell Cycle.

[120] Alexandrova E.M., Yallowitz A.R., Li D., Xu S., Schulz R., Proia D.A., Lozano G., Dobbelstein M., Moll U.M. (2015). Improving survival by exploiting tumour dependence on stabilized mutant p53 for treatment. Nature.

[121] Schulz-Heddergott R., Stark N., Edmunds S.J., Li J., Conradi L.C., Bohnenberger H., Ceteci F., Greten F.R., Dobbelstein M., Moll U.M. (2018). Therapeutic Ablation of Gain-of-Function Mutant p53 in Colorectal Cancer Inhibits Stat3-Mediated Tumor Growth and Invasion. Cancer Cell.

[122] Wang Y., Suh Y.A., Fuller M.Y., Jackson J.G., Xiong S., Terzian T., Quintas-Cardama A., Bankson J.A., El-Naggar A.K., Lozano G. (2011). Restoring expression of wild-type p53 suppresses tumor growth but does not cause tumor regression in mice with a p53 missense mutation. J. Clin. Investig..

[123] Christophorou M.A., Martin-Zanca D., Soucek L., Lawlor E.R., Brown-Swigart L., Verschuren E.W., Evan G.I. (2005). Temporal dissection of p53 function in vitro and in vivo. Nat. Genet..

[124] Bykov V.J., Issaeva N., Shilov A., Hultcrantz M., Pugacheva E., Chumakov P., Bergman J., Wiman K.G., Selivanova G. (2002). Restoration of the tumor suppressor function to mutant p53 by a low-molecular-weight compound. Nat. Med..

[125] Yu X., Vazquez A., Levine A.J., Carpizo D.R. (2012). Allele-specific p53 mutant reactivation. Cancer Cell.

[126] Lambert J.M., Moshfegh A., Hainaut P., Wiman K.G., Bykov V.J. (2010). Mutant p53 reactivation by PRIMA-1MET induces multiple signaling pathways converging on apoptosis. Oncogene.

[127] Lambert J.M., Gorzov P., Veprintsev D.B., Soderqvist M., Segerback D., Bergman J., Fersht A.R., Hainaut P., Wiman K.G., Bykov V.J. (2009). PRIMA-1 reactivates mutant p53 by covalent binding to the core domain. Cancer Cell.

[128] Li D., Marchenko N.D., Schulz R., Fischer V., Velasco-Hernandez T., Talos F., Moll U.M. (2011). Functional inactivation of endogenous MDM2 and CHIP by HSP90 causes aberrant stabilization of mutant p53 in human cancer cells. Mol. Cancer Res..

[129] Alexandrova E.M., Moll U.M. (2017). Depleting stabilized GOF mutant p53 proteins by inhibiting molecular folding chaperones: A new promise in cancer therapy. Cell Death Differ..

[130] Li D., Marchenko N.D., Moll U.M. (2011). SAHA shows preferential cytotoxicity in mutant p53 cancer cells by destabilizing mutant p53 through inhibition of the HDAC6-Hsp90 chaperone axis. Cell Death Differ..

[131] Parrales A., Ranjan A., Iyer S.V., Padhye S., Weir S.J., Roy A., Iwakuma T. (2016). DNAJA1 controls the fate of misfolded mutant p53 through the mevalonate pathway. Nat. Cell Biol..

[132] Zhang C., Liu J., Liang Y., Wu R., Zhao Y., Hong X., Lin M., Yu H., Liu L., Levine A.J. (2013). Tumour-associated mutant p53 drives the Warburg effect. Nat. Commun..

[133] Gupta A., Shah K., Oza M.J., Behl T. (2019). Reactivation of p53 gene by MDM2 inhibitors: A novel therapy for cancer treatment. Biomed. Pharm..

[134] Lane D.P., Cheok C.F., Lain S. (2010). p53-based cancer therapy. Cold Spring Harb. Perspect. Biol..

[135] Levine A.J. (2018). Reviewing the future of the P53 field. Cell Death Differ..

